# Acute fulminant invasive pulmonary aspergillosis in an immunocompetent host: An autopsy case report

**DOI:** 10.1016/j.mmcr.2018.02.002

**Published:** 2018-02-09

**Authors:** Yuuki Ohara, Takahiko Ito, Makoto Ito, Kyoko Yamashita, Shinya Toyokuni

**Affiliations:** a Department of Pathology and Biological Responses, Nagoya University Graduate School of Medicine, 65 Tsurumai-cho, Showa-ku, Nagoya 466-8550, Japan; b Department of Hematology, JCHO Kani Tono Hospital, 1221-5 Dota, Kani 509-0206, Japan; c Department of Pathology and Laboratory Medicine, Kariya Toyota General Hospital, 5-15 Sumiyoshi-cho, Kariya 448-0852, Japan

**Keywords:** Invasive pulmonary aspergillosis, *Aspergillus fumigatus*, Immunocompetent host, Smoker, Welder, Alcoholism

## Abstract

A 62-year-old previously healthy male who was a welder/smoker/drinker was admitted to Kani Tono Hospital for severe hypoxemia (Day 0). Initial physical and radiological examinations suggested an acute exacerbation of chronic obstructive pulmonary disease. However, respiratory failure developed rapidly, and he died on Day + 4. *Aspergillus fumigatus* was identified after his death, and he was diagnosed with invasive pulmonary aspergillosis. The clinical and pathological features are precisely described with pathogenetic considerations.

## 1 Introduction

Invasive pulmonary aspergillosis (IPA) is generally seen in immunocompromised hosts. Risk factors include malignant tumors, organ transplant, HIV infection, chemotherapy, diabetes mellitus, and corticosteroid therapy [1], [2]. Although the *Aspergillus*-specific galactomannan test and a timely radiographic screening can effectively diagnose IPA, overall mortality rates remain high despite antifungal treatments [1]. In addition to opportunistic infections, acute community-acquired IPA should be recognized as an uncommon form of invasive aspergillosis. Several local and systemic risk factors are mutually involved, including malnourishment, chronic obstructive pulmonary disease (COPD), and alcoholism [1], [2], [3], [4]. Certain viral respiratory infections, such as the influenza virus, may also predispose patients to this invasive fungal infection [1], [4], [5], [6].

Herein, we report an autopsy case of fulminant IPA in a previously healthy male. Although IPA is rarely encountered in healthy individuals, our case exacerbated rapidly. Of note, acute community-acquired necrotizing pneumonia is triggered by potentially virulent fungal pathogens, especially *Aspergillus* species, on rare occasions.

## 2 Case

**Fig. 1:**
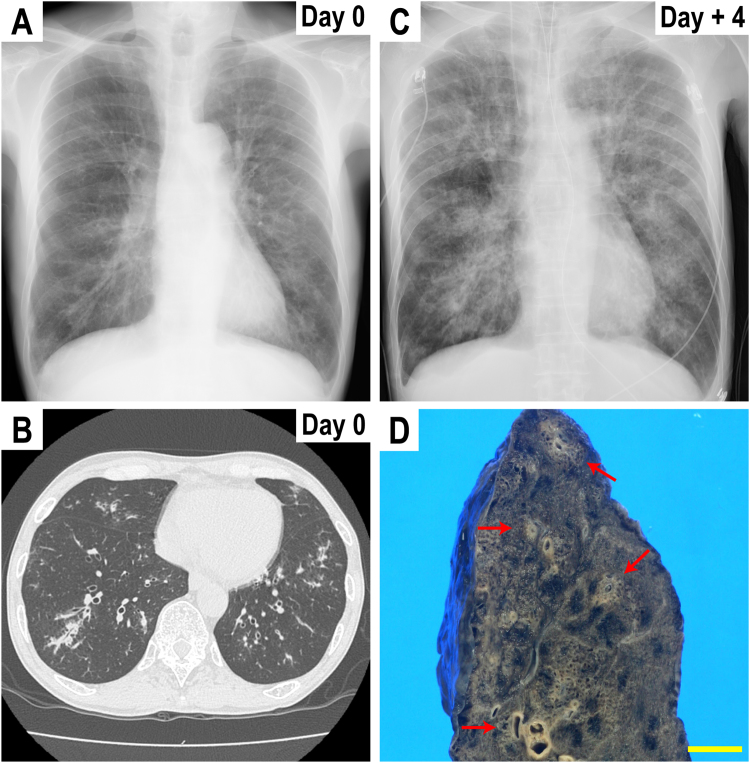
**Radiological images and macroscopic findings.** (A) Chest radiograph upon admission (Day 0, standing position). (B) Thoracic CT (Day 0) showing a slight thickening of the bronchial walls. (C) Chest radiograph (Day + 4, decubitus position) showing an infiltrative shadow. (D) Macroscopic findings of the lung at autopsy (Day + 4). Most bronchi are surrounded by ocher membranous lesions (arrows). Scale bar, 10 mm.

A 62-year-old male was referred to the outpatient clinic of Kani Tono Hospital for a fever lasting seven days and suspected pneumonia. He had no medical, medication, or family history. The patient was a current cigarette smoker (40 cigarettes per day) and a habitual drinker consuming 42–84 g of ethanol per day. His occupation was a welder, presumably exposed to metallic dust. His vital signs at admission were as follows: body temperature, 37.8 °C; pulse rate, 120 bpm; blood pressure, 138/81 mmHg; and SpO_2,_ 83% in room air. Physical examination showed a dry cough, dyspnea, tachypnea, and no pretibial edema. Bilateral wheezing was auscultated. Laboratory data showed a white blood cell count of 10,600 cells/μL (neutrophils, 86%; lymphocytes, 5%; monocytes, 10%; eosinophils, 0%; basophils, 0%); hemoglobin (Hb), 15.0 g/dL; HbA1c, 5.5%; platelets, 154,000/μL; and C-reactive protein, 22.39 mg/dL. Hepatic and renal functions were within normal limits. Tests were negative for human immunodeficiency virus, hepatitis B virus, and hepatitis C virus. Arterial blood gases were pH, 7.459; pO_2_, 53.4 mmHg; pCO_2_, 40.6 mmHg; HCO_3_^-^, 28.4 mEq/L; base excess, 4.6 mEq/L; lactate, 18.0 mg/dL; and blood glucose, 147 mg/dL. Chest radiography showed a slight infiltrative shadow (Fig. 1A). Thoracic computed tomography (CT) showed non-specific findings, such as thickened bronchial walls (Fig. 1B), slightly increased bronchial density, and mild emphysematous changes. The patient was immediately hospitalized (Day 0), suspected of an acute COPD exacerbation. Treatment started with the following drugs: 6-methylprednisolone, 40 mg ×2; ceftriaxone, 2 g; garenoxacin, 400 mg; and salmeterol xinafoate/fluticasone propionate.

On Day + 2, his respiratory status became severely dyspneic, and his arterial blood gases were pH, 7.25; pO_2_, 82.5 mmHg; pCO_2_, 78.0 mmHg; and HCO_3_^-^, 33.0 mEq/L. Noninvasive positive-pressure ventilation was performed, alternatively with an oxygen mask.

Invasive positive-pressure ventilation with tracheal intubation was started on Day + 3 due to exacerbated respiratory failure. However, effective oxygenation was not achieved because decreased pulmonary compliance and increased airway resistance made efficient ventilation difficult. Arterial blood gases were pH, 7.08; pO_2_, 70.9 mmHg; pCO_2_, 116 mmHg; and HCO_3_^-^, 32.7 mEq/L. The patient also developed heart and renal failure (BNP, 1543.5 pg/mL; BUN, 53.7 mg/dL; and creatinine, 2.92 mg/dL).

On Day + 4, a chest radiograph showed a clear infiltrative shadow (Fig. 1C). His platelet count was reduced to 88,000/μL, with D-dimer concentrations being elevated to 77.9 μg/mL. The overall data represented systemic inflammatory response syndrome with coagulative disorder. A filamentous fungus was isolated from the sputum collected on Day + 2. His serum (1,3)-β-D-glucan level was elevated to 530.7 pg/mL. Preemptive antifungal treatment was initiated at 70 mg of caspofungin acetate intravenously. However, therapeutic interventions did not improve the multiple organ failure, and the patient died on Day + 4. Three days later (Day + 7), the isolated fungus was identified as *Aspergillus fumigatus* based on colony morphology.

**Fig. 2:**
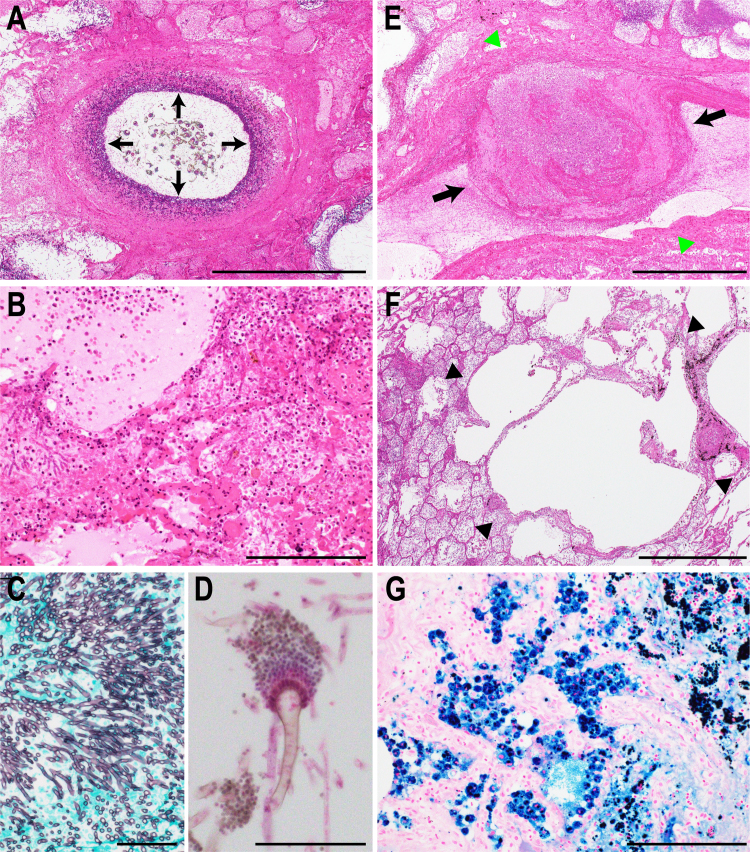
**Histological findings.** (A) Exudative inflammation around the bronchus, corresponding to the ocher membranous lesions in Fig. 1D. *Aspergillus* (arrows) proliferation in a bronchus. (B) Severe alveolar damage. The alveoli show necrosis, with neutrophil infiltration, hemorrhaging, and edema. (C) *Aspergillus* mycelia stained with Grocott's methenamine silver. (D). Conidial heads of *Aspergillus*. (E) Thrombosis. A thrombus (arrows) is seen in the blood vessel (arrow heads). (F) Low power magnification of the lung tissue. Emphysematous changes in alveoli (arrow heads) are not severe. (G) Iron deposition in the lung. Macrophages phagocytizing iron shown by positive Berlin blue staining. (A), (B), (D)-(F) H&E staining, (C) Grocott's methenamine silver staining, (G) Berlin blue staining. Scale bar, 1 mm in (A), (E) and (F); 200 µm in (B) and (G); 50 µm in (C) and (D).

An autopsy was performed three hours after the patient's death (Day + 4). The body height was 160 cm, and the weight was 50 kg. The most remarkable findings were confined to the lungs. Grossly, both lungs were swollen and profusely consolidated. From the main bronchus to the peripheral branches, most bronchi were surrounded and occluded by necrotic ocher membranous lesions (Fig. 1D). A diffuse pulmonary hemorrhage was also observed. Histologically, the lung specimens revealed abundant *Aspergillus* proliferation in the bronchi, with severe necrosis and exudative inflammation (Fig. 2A). The microscopic bronchial lesions corresponded to the macroscopic ocher membranous lesions (Fig. 1D). *Aspergillus* also invaded the alveolar areas. These areas were severely damaged, showing neutrophil infiltration, hemorrhaging, and edema (Fig. 2B). Grocott's methenamine silver staining clearly showed invasive mycelial fungal growth (Fig. 2C) as well as conidial heads (Fig. 2D). Angioinvasion was also demonstrated, with the affected vessels being obliterated by thrombi (Fig. 2E). Based on the sputum culture and these histological findings, IPA was diagnosed. Apart from invasive aspergillosis, alveolar emphysematous changes were focal and mild (Fig. 2F). Iron deposition inside the alveolar macrophages (hemosiderin-laden macrophages) was remarkable (Fig. 2G), a finding likely associated with the patient's job as a welder. Systemic examination showed no disseminated infection elsewhere. Notably, no predisposing cavity was found that would provoke a fungal colonization. No hepatic or pancreatic lesions from alcohol consumption were observed. Passive congestion and hyperemia were observed in several organs, including the liver, kidneys, and alimentary tract.

## 3 Discussion

IPA usually occurs in immunocompromised patients. In rare instances, however, it may affect immunocompetent hosts [1], [2], [3], [4] even with no predisposing non-invasive aspergillosis, such as simple aspergilloma or chronic cavity pulmonary aspergillosis. The present case was unique in that acute IPA developed into rapidly progressive respiratory failure. No clinical or radiographic evidence indicated a precedent fungal colonization. At the initial stage, it was difficult to consider a community-acquired pulmonary fungal infection.

Autopsy revealed angioinvasive aspergillosis extensively involving the entire lungs associated with necrotizing pneumonia and bronchitis. Anatomically, the patient's severe ventilation failure may have been related to the damaged bronchial structure collapsing along with massive intraalveolar exudate. The severe pneumonia could have led to decreased pulmonary compliance and increased pulmonary shunt. The membranous bronchitis is thought to have increased airway resistance. Thrombosis in pulmonary vessels and hemorrhaging would have caused ventilation/perfusion mismatch and pulmonary hypertension. These pathogenic events would result in ventilatory insufficiency (hypercapnic respiratory failure) progressing in the short term, despite using a mechanical ventilator, ultimately leading to multiple organ failure and death. In addition, the hypoxic state may have potentiated the invasive *Aspergillus fumigatus* growth. Recent studies have shown that *Aspergillus fumigatus* virulence depends highly on adaptability to a hypoxic microenvironment in the infective site [7]. In a severely hypoxic state, *Aspergillus fumigatus* can survive and facilitate mycelial growth to vigorously invade the lung tissue as demonstrated in this case.

Several pulmonary conditions should be considered potential risk factors for certain invasive bacterial or fungal pathogens. IPA cases in immunocompetent patients with COPD have been reported [1]. According to the Japanese Guideline for Management of Deep-seated Mycoses 2014 [2], COPD and pulmonary fibrosis are listed as predisposing factors for IPA. The present patient was a longstanding cigarette smoker and a welder believed to be constantly exposed to metallic dust. Cigarette smoke and/or a deficiency of surfactant proteins in COPD can cause dysfunction in immune response against pathogens, including fungi [8], [9], [10]. Welder's siderosis (arc welder's pneumoconiosis) is caused by iron oxide accumulating inside alveolar macrophages [11]. Although welder's siderosis does not cause pulmonary fibrosis and typically produces no symptoms, silicosiderosis can occur and pulmonary fibrosis may develop when a substantial quantity of silica mixes with the inhaled iron [11]. In addition, iron overload has also been linked to an increased risk of bacterial [12] and fungal infections independently of IPA in immunocompromised patients [13], [14]. Iron overload catalyzes the Fenton reaction, generating hydroxyl radicals, which damage the body and play important roles in various lesions, including fibrosis [15] and tumors [16], [17]. In the present case, no severe COPD or pulmonary fibrosis was observed. However, the iron deposition and locally damaged airway condition with the impaired mucociliary clearance by cigarette smoke may have contributed to the fungal infection. Because *Aspergillus* was localized in the lungs (without disseminated infection), iron overload was suspected to be involved with the *Aspergillus* infection in the present case.

Based on CT (Fig. 1B) and autopsy (Fig. 1D) findings, the patient may have been suffering from *Aspergillus* tracheobronchitis (ATB) at admission, which had progressed to IPA when he died. ATB is a rare, confined form of IPA [18] and could be an early stage of IPA [19]. Non-specific radiographic findings make it difficult to diagnose. In COPD patients, ATB symptoms would likely be regarded as COPD exacerbations [20].

Certain respiratory viral infections are said to involve risks for developing IPA [1], [4], [5], [6]. Unfortunately, no data relevant to an influenza infection was available in the present case.

In the case reported here, the patient was a smoker, a heavy alcohol drinker, and a welder. The combination of these risk factors would promote *Aspergillus* infection and rapid IPA development.
